# New boost type single phase inverters for photovoltaic applications with reduced device count

**DOI:** 10.1371/journal.pone.0304463

**Published:** 2024-07-12

**Authors:** Zuhair Muhammed Alaas

**Affiliations:** Electrical and Electronics Engineering Department, College of Engineering and Computer Sciences, Jazan University, Jazan, Saudi Arabia; Ghani Khan Choudhury Institute of Engineering and Technology, INDIA

## Abstract

In recent years, single-stage boost inverters with common ground have shaped the inverter markets due to the many benefits associated with these types of inverters, including their high efficiency, single control scheme, and integrated boost converter. A new boost-type inverter that utilizes a common ground and has fewer switches is proposed in this article. It uses two DC-link capacitors connected in parallel and discharged independently while being charged simultaneously. The voltage for the positive and negative half cycles is supplied by the capacitors located at the top and bottom of the circuit, respectively. In addition, a comparison is made between the proposed circuit and the boost inverter already in use in the literature. Using PLECS as the computing software, the efficiencies are determined depending on the various percentages of output power. To validate performance, present experimental data, and attain the best possible efficiency of 97%, a 400 W prototype model is constructed. In addition to that, the breakdown of the costs is shown.

## Introduction

In grid-connected transformer-less photovoltaic (PV) inverters, leakage current is one of the most important problems that might arise. This is because of the high-frequency common-mode voltage and the potential-induced deterioration (PID) polarization effect [1]. For single-phase applications, the conventionally available two-level full-bridge inverter is the most common type of photovoltaic inverter employed. Common mode voltage and leakage current, on the other hand, provide substantial challenges [2–4].

Recent studies reveal that the common ground type (CGT) inverter could suppress the leakage current due to the direct connection between the grid and the PV panel’s negative terminals. However, several single-stage transformer-less CGT-based inverter topologies are well reported in [5–7]. To develop the inverter with its four switches, the two boost converters must first be linked at each end of the DC source terminals [6].

In addition, this topology requires two inductors, which, as a result of the predetermined dead time, causes the production of a greater amount of electromagnetic interference. By replacing the inductor with a coupled inductor, the topology explained in [7] improves the structure presented in [6]. A high voltage dependent on the turn ratio is generated by the coupled inductor, which also serves to dampen the output ripples. However, the same issue persists here, which makes tracking the maximum power point in PV applications even more difficult. The integrated boost and full bridge inverter structures are presented in [8]. Although this topology eliminates cross-over distortion, it suffers from high voltage stress on the DC-link capacitor and switching loss of full bridge inverters. A new half-bridge inverter-based topology with the integration of a boost converter is presented in [9], and this topology has one high switching frequency switch for each half cycle. Although the topologies described above are appropriate for PV applications, the leakage current is still a little high. Further, the CGT is a good choice to suppress the PID effect.

**Fig 1:**
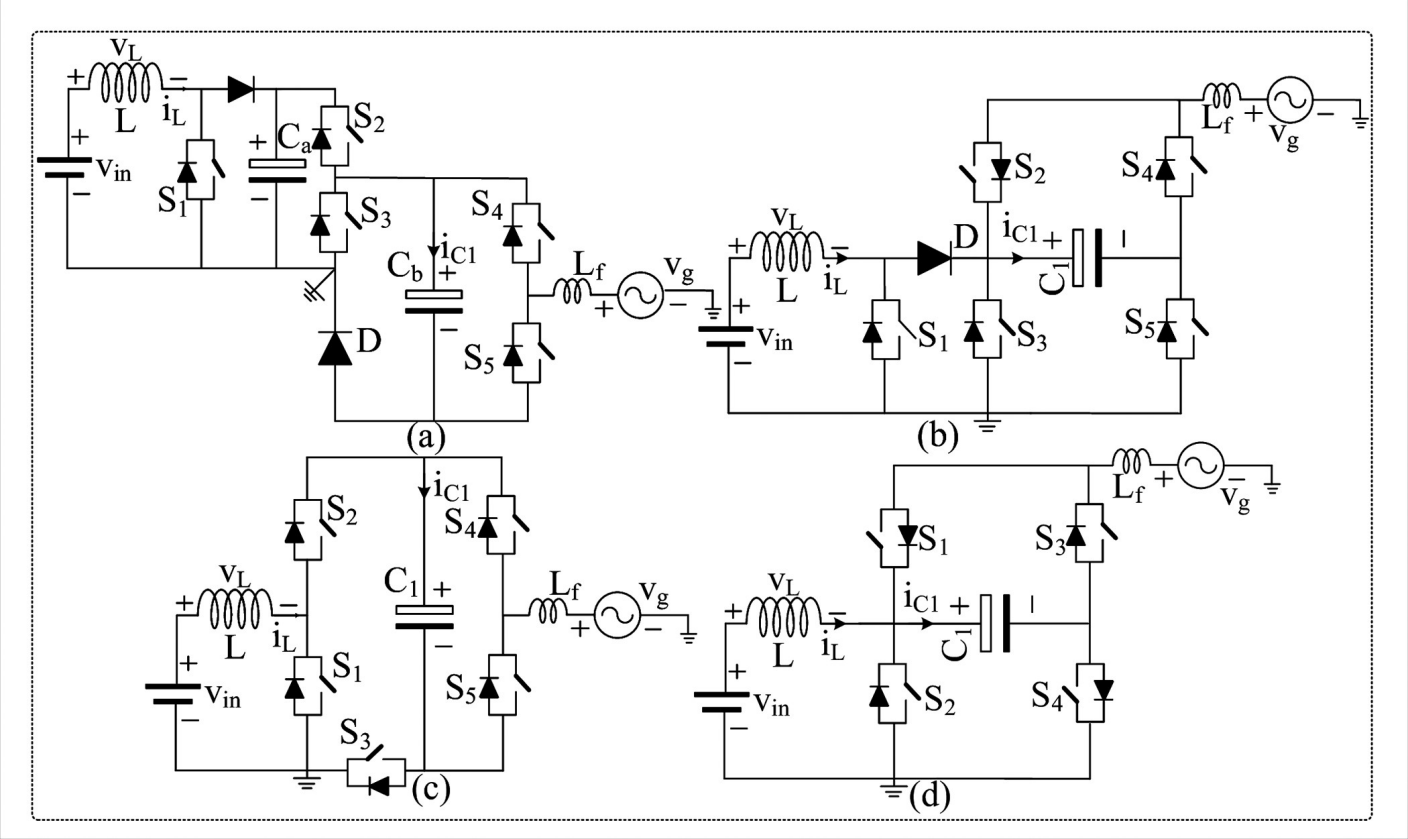
Existing H-bridge derived CGT boost Inverters: (a) structure presented in [10], (b) structure presented in [11,12], (c) structure presented. in [13], and (d) structure presented in [14].

Fig 1(A)–1(D) depicts the well-known three-level inverter topologies with a common ground feature. The common ground is achieved by inserting an additional capacitor (acting as a virtual DC source) in the H-bridge inverter shown in Fig 1(A) from [10]. Further, this topology does not have the boosting ability, and the front-end DC-DC converter is included in the circuit to boost the input voltage. In addition, the presented DC-DC converter suppresses the inrush current by using the boost converter inductor, called the soft charging method. The topology depicted in Fig 1(B) and described in [11,12] is an improved version of the structure given in [10] with one capacitor and diode removed.

Nevertheless, the topologies [11,12] have the same structure, except that the structure given in [10] cannot feed reactive power into the grid, and the capacitor acts as a virtual DC source. By eliminating the front-end DC-DC converter and the inductor, an integrated H-bridge inverter with five switches is presented in [13], as shown in Fig 1(C). The boosting inductor is positioned in such a way as to produce boosting voltage with the assistance of one additional switch. As reported in [14], a recently proposed CGT topology with four switches and one capacitor lacks a boosting feature, as depicted in Fig 1(D). In addition, this topology requires a capacitor with a voltage rating three times that of the grid voltage and a high inductance value. Further, the input voltage must correspond to the grid voltage. However, the previously stated single-phase, single, and double-stage inverter topologies have garnered substantial attention, and research and the development of novel techniques are ongoing.

In this regard, this article proposes a new single-stage boost inverter with common ground. The proposed topology provides a low leakage current with the same components as a conventional two-stage single-phase inverter. It uses a pair of parallel-connected DC-link capacitors that are charged simultaneously and discharged independently. The capacitors at the top and bottom of the circuit, respectively, supply the voltage for the positive and negative half cycles. Furthermore, a comparison is presented between the boost inverter that is currently in use in the literature and the suggested circuit. PLECS (a software tool for system-level simulations of electrical and electronic circuits) calculates the efficiency based on different output power percentages. A 400 W prototype model is constructed to verify performance, show experimental results, and achieve the highest possible efficiency of 97%. Furthermore, the breakdown of costs is provided.

The rest of the manuscript is organized as follows: Section 2 presents the proposed boost inverter, a description of the operating principle, and its pulse generation scheme. Section 3 presents an analysis of the power loss calculation. The proposed scheme is compared with more modern transformer-less three-level inverter topologies in Section 4. Section 5 shows that the proposed topology can be extended for a common ground connection for two DC sources. In Section 6, simulation and experimental results and discussion are provided. Section 7 concludes the work presented.

## Proposed three-level boost inverter

### 2.1 Description of the topology proposed

**Fig 2:**
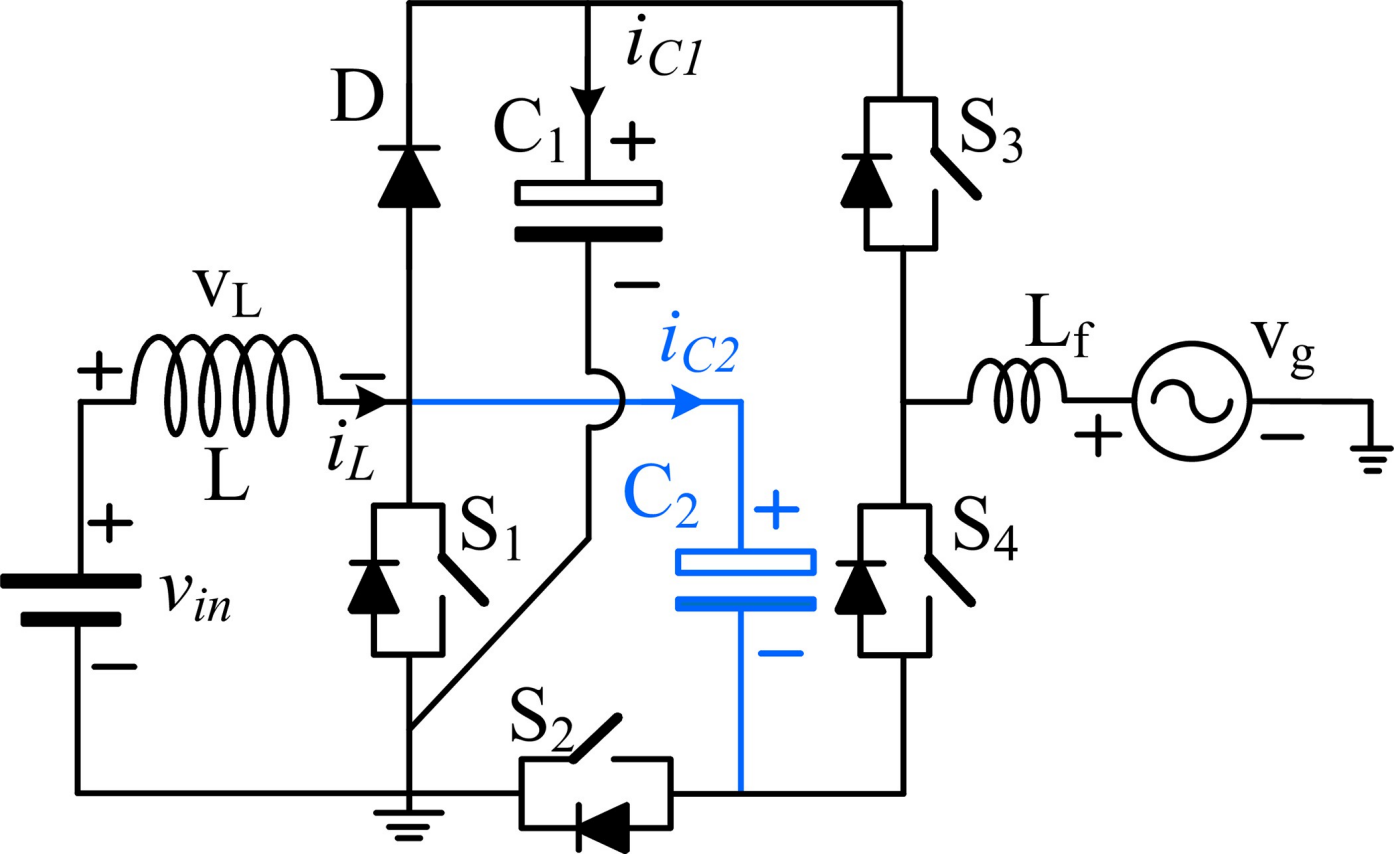
The proposed three-level common ground type inverter topology.

Fig 2 depicts the circuit diagram of the proposed topology, which consists of two capacitors, four switches, one diode, and one inductor. One end of the inductor is connected to the source voltage (*v*_in_), and the other is connected between the diode anode and the drain terminal. As depicted in Fig 2, the capacitor (*C*_1_) is connected to the cathode of the diode, while the other end is connected to the switch *S*_1_ source terminal. The capacitor (*C*_2_) is connected between the switches *S*_1_ and *S*_2_. The switches *S*_3_ and *S*_4_ form the half-bridge module, and both are turned on and off in a complementary manner.

### 2.2 Principle of operation

**Fig 3:**
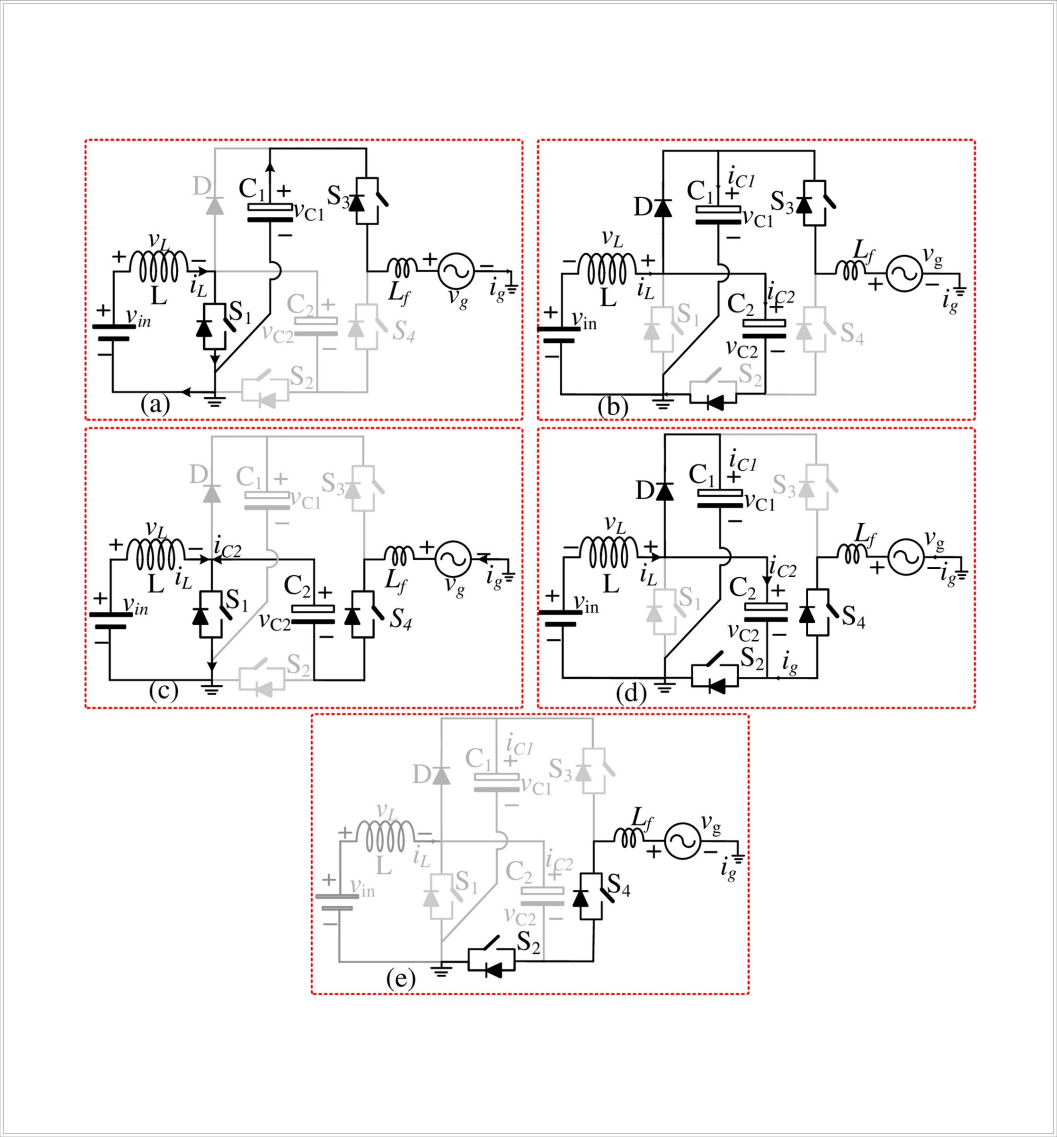
Modes of operation of the proposed type topology: (a) mode 1, (b) mode 2, (c) mode 3, (d) mode 4 and (e) mode 5.

Fig 3 shows the five modes of operation of the proposed topology. They are described as follows:

In mode +*v*_*C1*_, shown in Fig 3(A), the switch *S*_1_ is closed, and the capacitor *C*_1_ supplies the load through the switch *S*_3_. The load output equation is:

*v*_*o*_ = *v*_*C*1_ and the inductor is charging as given in (1):

$$L\frac{d_{iL}}{dt}=v_{in}-(r+R_{x})i_{L},$$

$$C_{1}\frac{d_{v_{C1}}}{dt}=-\frac{v_{C1}}{R},$$

$$C_{2}\frac{d_{v_{C2}}}{dt}=0.$$ (1)

In mode (*+v*_*in*_*+v*_*L*_), shown in Fig 3(B), the switch *S*_1_ is open, and the inductor current charges the capacitors *C*_1_ and *C*_2_ and supplies the load through switch *S*_3_. The load output equation is *v*_*o*_ = +*v*_*m*_+*v*_*L*_, and the inductor is charging, as expressed in (2).


$$L\frac{d_{i_{L}}}{dt}=v_{in}-v_{C1,2},$$

$$C_{1}\frac{d_{v_{C1}}}{dt}=i_{L}-(\frac{v_{C1}}{R}),$$

$$C_{2}\frac{d_{v_{C2}}}{dt}=0.$$ (2)

In mode (-*v*_*C2*_), shown in Fig 3(C), the switch *S*_1_ is closed, and the capacitor *C*_2_ supplies the load through switch *S*_4_. The load output equation is *v*_*o*_ = *v*_*C*2_, and the inductor is charging, as given in (3).


$$L\frac{d_{i_{L}}}{dt}=v_{in}-(r+R_{x})i_{L},$$

$$C_{1}\frac{d_{v_{C1}}}{dt}=0,$$

$$C_{2}\frac{d_{v_{C2}}}{dt}=-\frac{v_{C2}}{R}.$$ (3)

In modes (*freewheeling*) and (*+0 v*_*in*_), shown in Fig 3(D)-3(E), the switch *S*_1_ is open, and the inductor current charges the capacitors *C*_1_ and *C*_2_, and the switch *S*_2_ will be conducted to provide the freewheeling path for the load. The load output equation is *v*_*o*_ = 0, and the inductor is charging, as given in (4).


$$L\frac{d_{i_{L}}}{dt}=v_{in}-v_{C1,2},$$

$$C_{1}\frac{d_{v_{C1}}}{dt}=C_{2}\frac{d_{v_{C2}}}{dt}=i_{L}.$$ (4)

However, in these modes, the output voltage will be zero. Table 1 summarizes the switching sequence of the proposed boost inverter.

**Table 1:** Switching sequence of the proposed boost inverter.

Modes	Switches for type I/II	*L*	Capacitor		*v*_*g*_
			*C*_1_	*C*_2_	
1	*S*_1_ on, and *S*_3_ on	Charging	Discharging	-	+*v*_*C1*_
2	*S*_1_ off, and *S*_3_ on	Discharging	Charging	Charging	+*v*_in_+*v*_L_
3	*S*_1_ on, and *S*_4_ on	Charging	-	Discharging	−*v*_*C2*_
4	*S*_4_ on, and *S*_2_ on	Discharging	Charging	Charging	Freewheeling path
5	*S*_4_ on, and *S*_2_ on	-	-	-	0 *v*_*in*_

The voltage boost factor of the proposed inverter relies on the duty cycle of the switch *S*_1_, which is determined by (5).


D=M|sin(ωt)| (5)

The voltage across the DC-link capacitors, *C*_1_=*C*_2_, is derived as follows:

$$v_{C1}=v_{C2}=\frac{v_{in}}{1-M|\sin(\omega t)|}$$ (6)

The calculated minimum capacitance of the capacitors primarily depends on the maximum power transfer, *v*_o_, switching frequency, and the maximum allowable capacitor ripple voltage. The approximate value of the capacitors can be obtained by multiplying the calculated minimum capacitance by the operating frequency as given in (7).

$$C_{1},C_{2}>=\frac{P_{o}}{\Delta v_{C}v_{o}f_{sw}}$$ (7)

where *P*_*o*_ is the output power and *f*_sw_ is the switching frequency. Due to the capacitors’ low equivalent series resistance, inverter operation and experimental validation typically require a higher capacitor value than the estimated one. Similarly, the inductor selection is the same as the boost converter, but it is designed in the DCM mode, and the critical inductance (*L*_*Cric*_) value is eight.

However, modest changes must be made in practical implementations. Because of the exceptionally low equivalent series resistance of the capacitors, the capacitor value should usually be more than the estimated value during inverter operation and experimental validation. Similarly, the selection of the inductor is identical to that of the boost converter. Still, it is designed in DCM mode, and the critical inductance (*L*_*Cric*_) value is calculated as follows:

$$L\leq L_{Cric}=\frac{M{v_{in}}^{2}}{4v_{o}f_{sw}}$$ (8)

### 2.3 Pulse generation scheme

**Fig 4:**
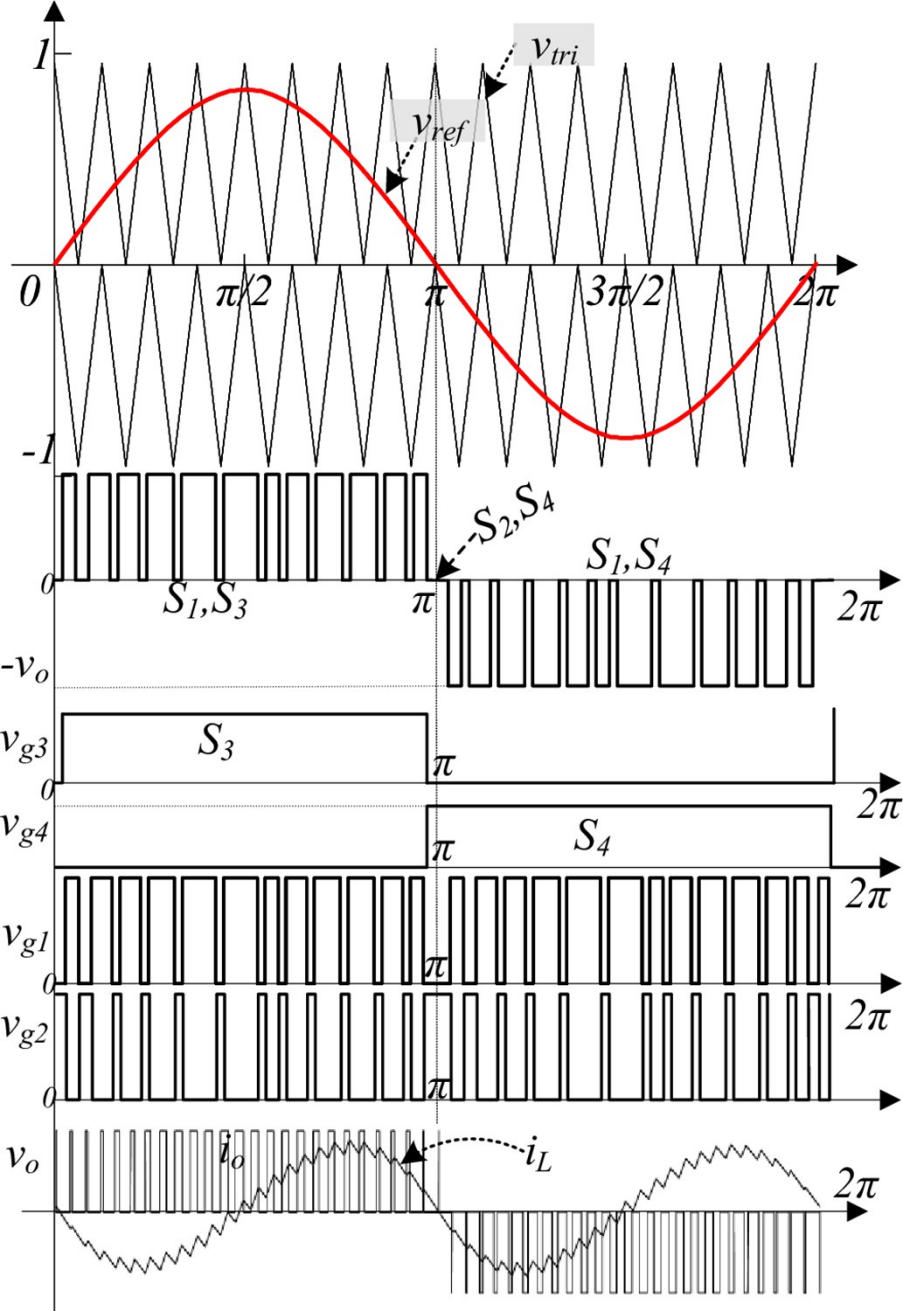
Typical two-level output voltage waveform and gate pulses.

The pulse generation scheme for the proposed topology is shown in Fig 4. The level-shifted triangular pulse width modulation scheme is used (*v*_tri_) as a carrier signal compared with the reference signal (*v*_*ref*_) to generate the required pulses. During pulse generation, the switches *S*_3_ and *S*_4_ operate at the fundamental switching frequency, i.e., 50 Hz, but the switches *S*_1_ and *S*_2_ are complementary switches that operate at high frequency, i.e., 20 kHz. It confirms that the proposed topology does not require additional pulses to boost the input voltage since switches *S*_1_ and *S*_2_ are operated at a high frequency. The pulses are obtained as expressed in (9) and (10).


$$v_{g1}={v_{tri}}^{+}>v_{ref}+{v_{tri}}^{-}<v_{ref}$$ (9)

$$v_{g2}=∼v_{g1}$$ (10)

## Power loss analysis

**Fig 5:**
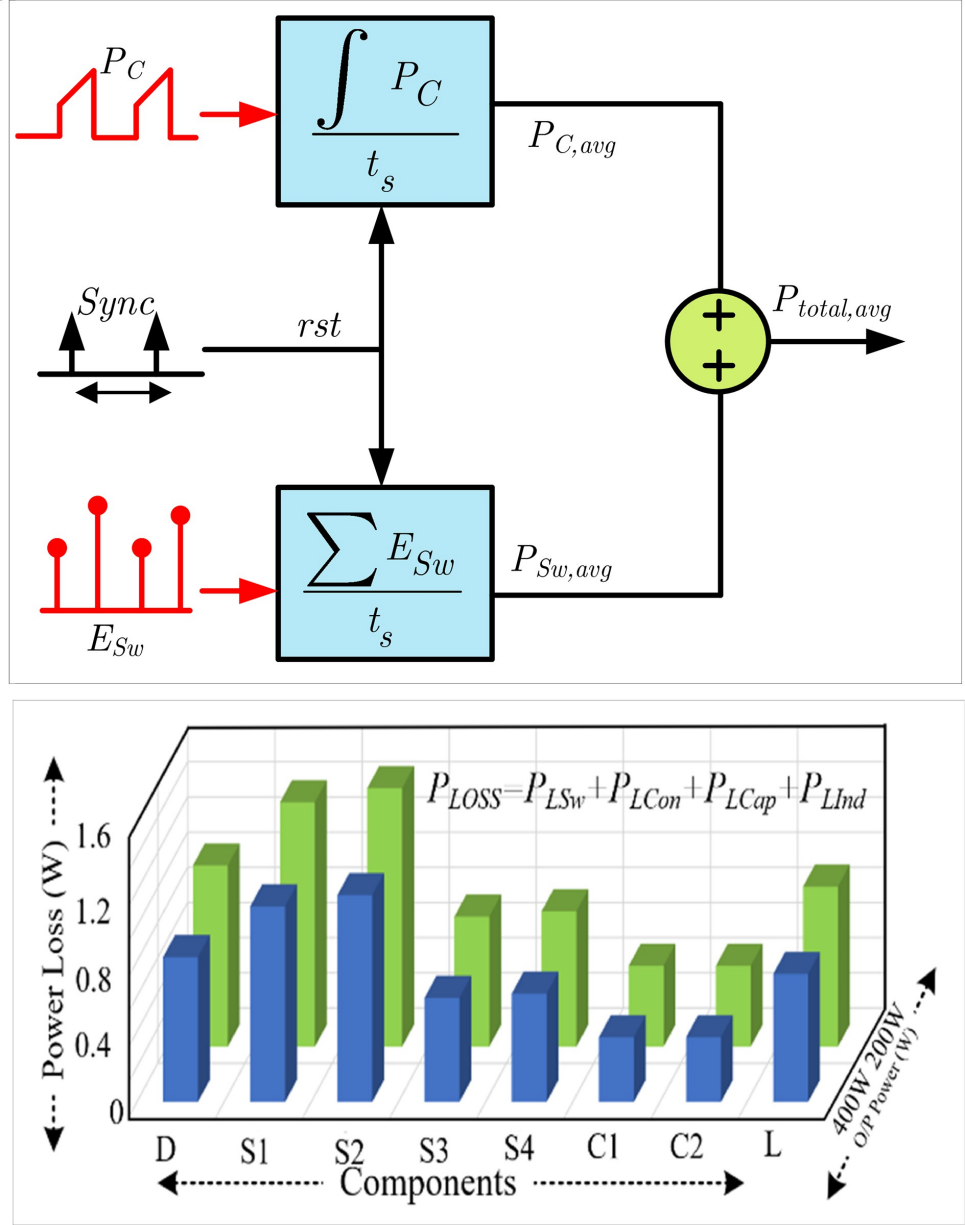
PLECS power loss calculation: (a) total cycle average losses calculation method and (b) power loss breakdown for the different components.

The switch, Infineon IGBT (IGB30N60T), with an anti-parallel diode switch datasheet, is used to calculate the power loss of the switches. The individual component’s power loss breakdown using the PLECS calculated is depicted in Fig 5(A). In the simulation, as discussed earlier, the capacitor loss is lower than the switching losses. Switches *S*_1_ and *S*_2_ have a higher loss due to operating at a higher switching frequency of 20 kHz, but switches *S*_3_ and *S*_4_ operate under fundamental switching frequency with low power losses.

Sensing the rising and dropping edges of the voltages throughout a simulation period (*t*_s_), the number of switching events *n* may be computed. Further, the peak current and voltage may also be monitored. The datasheet describes the switching energy required to determine the average turn-on and turn-off loss of the power switch, as shown in (11).


$$P_{Sw,avg}=\frac{1}{t_{s}}\sum_{k=1}^{n}E_{sw}=\frac{1}{t_{s}}\sum_{k=1}^{n}f_{Eon}(i_{peak}(n),v_{peak}(n))+f_{Eoff}\left(i_{peak}(n),v_{peak}(n)\right)$$ (11)

The current and peak voltage at the *n*th rising and falling voltage edges are i_peak_ (*n*) and v_peak_ (*n*), respectively. When the power switch is on and off, the peak voltage and current are the switching energy functions *f*_Eon_ and *f*_Eoff_. The power switch’ average conduction loss is given in (12).

$$P_{C,avg}=\frac{1}{t_{s}}\int_{0}^{t_{s}}P_{cond}=\frac{1}{t_{s}}\int_{0}^{t_{s}}i_{C}(t)\cdot v_{CE}(t)\cdot dt$$ (12)

where *i*_*c*_ and *v*_*CE*_ are the collector current and emitter-collector voltage, respectively. Fig 5(A) shows the schematic technique cycle-average loss computation, and Fig 5(B) shows the simulated component losses for 200 W and 400 W power output.

## Comparative study with modern transformer-less three-level inverter topologies

The proposed scheme is compared with more modern transformer-less three-level inverter topologies in Table 2. Reactive power control becomes harder with the topology presented in [4] due to the increasing number of components. Buck and boost are constructed utilizing more components in [5]. Additionally, the topology makes use of three inductors, which exacerbates the issue of electromagnetic interference (EMI). In [6], it is explained how to increase voltage with a common ground by using two capacitors and six switches. However, there is a commonality among the aforementioned topologies and an increasing number of passive or active devices. The dual boost inverter topology also referred to as the split inductor type-topology, was introduced in the studies presented in [7–9,15]. Comparing these topologies to the current topology, fewer components are used. Unfortunately, two magnetic components are needed, which exacerbates the EMI issue and power losses. A current presentation of the virtual DC source-based topology may be found in [10–12]. Except for the number of devices, these topologies functioned according to the same idea. Five switches are used in these common ground type topologies. Further, these topologies are separate from the single-stage operation, i.e., these topologies needed separate DC-DC boost converters to boost the input voltage. The single-stage boost inverter topology is presented in [13], where the boost converter is integrated with an h-bridge inverter. Still, this topology switch count is high compared to the proposed topology. In [14], the topology does not have a boosting ability. Even though one capacitor and one diode are additionally required in the proposed topology, the advantages are: (i) additional capacitor loss is less than the switching loss, and (ii) it does not require additional driver circuits.

**Table 2:** Component comparison between the proposed topology and different existing topologies*.

Ref.	*N*_*Sw*_	*N*_*Dri*_	*N*_*Di*_	*N*_*Cap*_	*N*_*Ind*_	*TC*	*BA*	*CMV*	*DG*	*P*_*o*_	*η*
[4]	5	5	3	1	1	14	Yes	No	Yes	193.2 W	--
[5]	5	5	2	2	3	17				400 VA	96.4%
[6]	6	6	2	2	2	18				400 VA	96.4%
[7]	4	4	-	2	2	12		Yes	No	500 W	--
[15]	4	4	-	2	2	12				260 W	97.5%
[8]	4	4	2	1	1	12				200 W	90.5%
[9]	4	4	3	2	1	14				100 W	90.0%
[10]	5	5	2	1	1	14		No	Yes	1000 VA	97.8%
[11]	5	5	1	1	1	13				200 W	96.0%
[12]	5	5	1	1	1	13				400 W	95.1%
[13]	5	5	-	1	1	12				440 W	95.5%
[14]	4	4	-	1	1	10	No			2000 W	96.0%
**Proposed**	**4**	**4**	**1**	**2**	**1**	**12**	**Yes**			**400 W**	**≈ 97.0%**

*BA, boosting ability; *CMV*, common mode voltage; DG, dual grounding; *N*_*Sw*_*/N*_*Dri*_*/N*_*Di*_ */N*_*Cap*_ */N*_*Ind*_, number of switches/driver circuits/ diodes/ capacitors/inductors; *TC*, total components; *P*_*o*_, output power; and *η*, efficiency.

## Extended dual source structure

**Fig 6:**
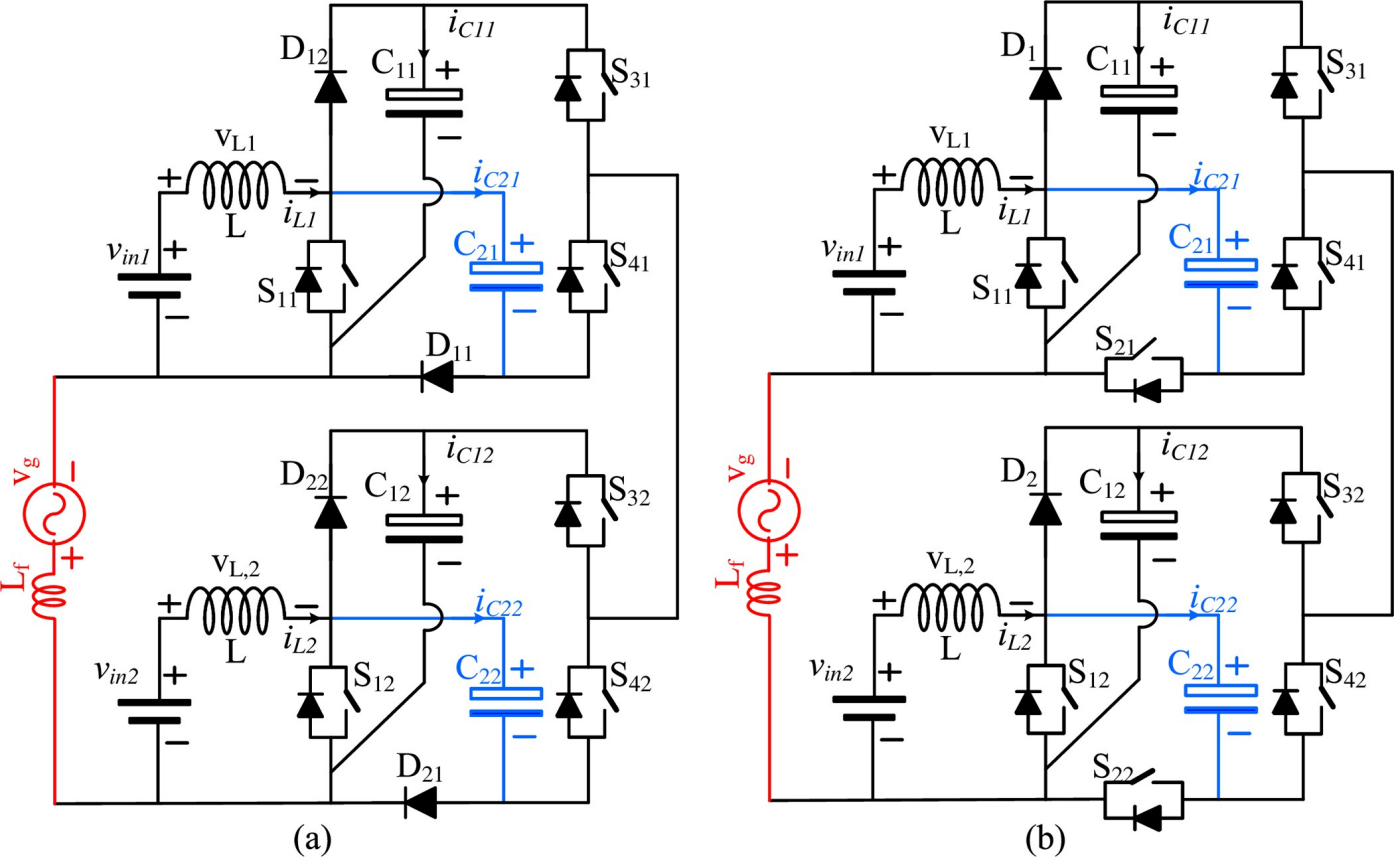
Extended structure of two DC sources with common ground.

The proposed topology can be extended for a common ground connection for two DC sources, as depicted in Fig 6(A) and 6(B). Both ends of the grid are connected to the negative terminals of the two DC sources. Existing topologies in the literature introduced a single DC source connection and did not show source extensions. However, only two DC sources can be connected to a common ground in the proposed topology. If the number of DC sources increases, the common ground feature will remain the same for the two DC sources.

As depicted in Fig 6(A), the number of switches for each unit is reduced to three, and the zero state is achieved by connecting *S*_31_ and *S*_32_ since all capacitors will maintain the same voltage. If one of the sources fails, it will be difficult to obtain the zero state and regulate the voltage across the capacitor of the failed DC source unit; therefore, it is preferable to disconnect both sources from the grid if one source fails. To address this issue, the second configuration of Fig 6(B), which is an extension of the configuration shown in Fig 2, is recommended. Since the zero states can be reached by turning on the switches *S*_21_ and *S*_22_, in this configuration, if either source fails, the other DC source will maintain the DC link voltage by adjusting the boost switch’s duty cycle to match the grid voltage.

## Simulation and experimental results and discussion

**Fig 7:**
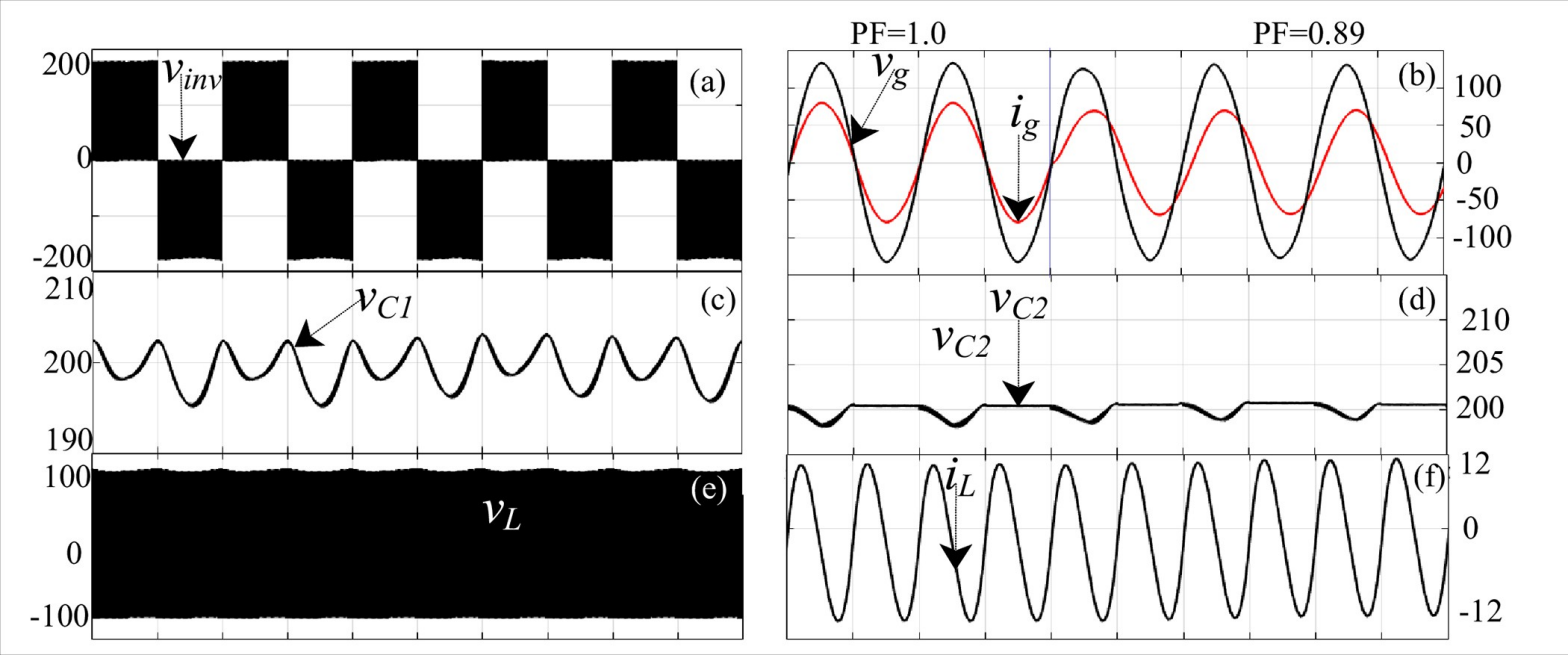
Simulation results: (a) inverter output voltage (*v*_*inv*_), (b) grid voltage (*v*_*g*_) and current (*i*_*g*_), (c) capacitor voltage (*v*_*C*1_), (d) capacitor voltage (*v*_*C*2_), (e) inductor voltage (*v*_*L*_), and (f) inductor current (*i*_*L*_).

Fig 7(A)–7(F) show the simulation results of the proposed topology where the input voltage is kept at 100 V, and the corresponding output voltage (*v*_o_) is 180 V (as shown in Fig 7(A)) for a modulation index of 0.8. The voltage across the capacitor voltage is 180 V, the voltage across the inductor is 80 V, and the sum of the source and inductor voltage is obtained as 180 V at the load terminals. A simple LCL filter provides a pure sinusoidal voltage to connect to the grid. As shown in Fig 7(B), the output voltage and current after the LCL filter with unity and 0.89 lagging power factor are presented. Since *C*_1_ and *C*_2_ are connected in parallel, the capacitor voltage will be the same, as shown in Fig 7(D). Further, the inductor voltage (*v*_*L*_) and currents (*i*_*L*_) are shown in Fig 7(E)-7(F), where *i*_*L*_ reaches a maximum of 12 A.

**Fig 8:**
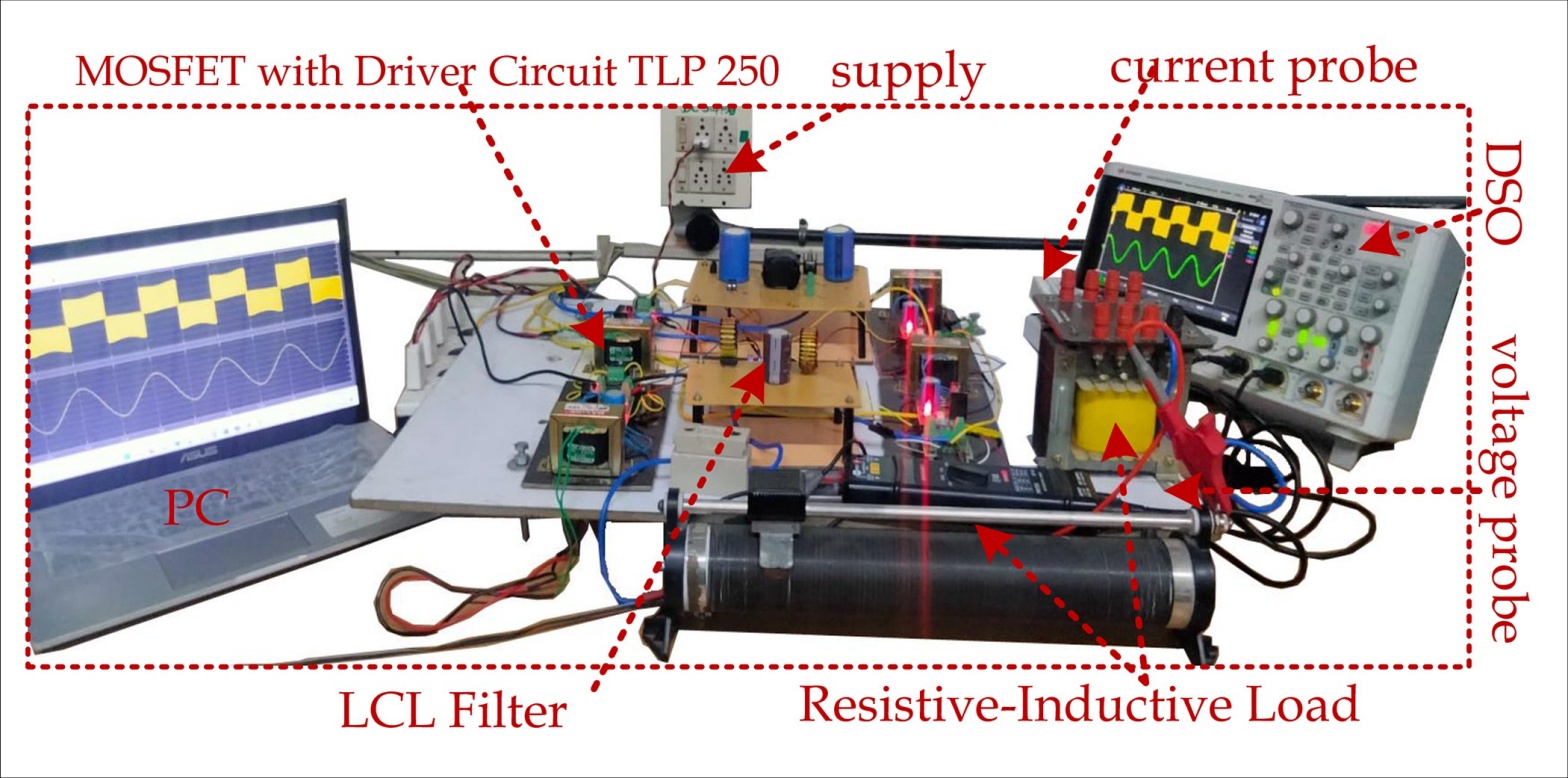
Hardware setup of the proposed topology.

Using the setup depicted in Fig 8, the performance of the proposed topology is validated. IGBT IKFW50N60ETXKSA1 is used with HCPL-316J-000E driver circuits in the experimental validation. The TMS320F28379D DSP controller, the ERLA211LIN172KA50M 250V/1700F capacitors, and an e-core type inductor is utilized. Initial testing of the proposed inverter involves a resistive-inductive load without the LCL filter. The input voltage is maintained at 100 V, and *R*=50 Ω, *L*=50 mH, and *R*=50 Ω, *L*=100 mH are utilized.

**Fig 9:**
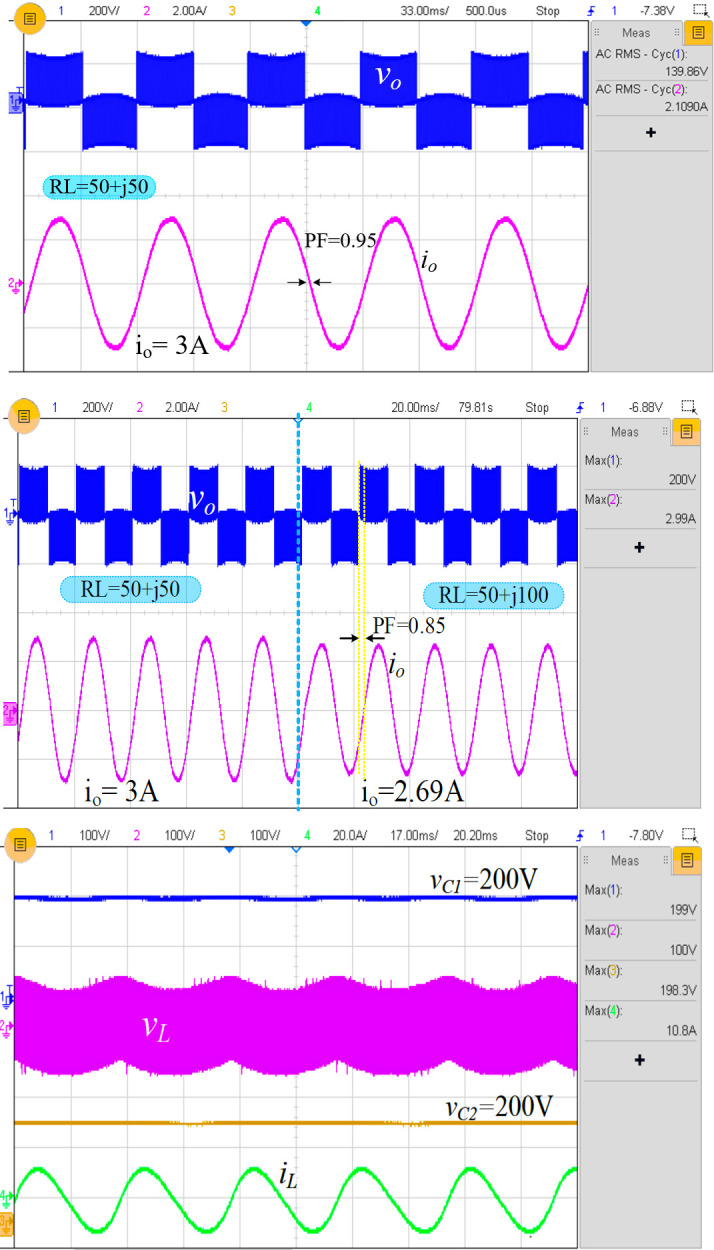
Experimental result: (a) *v*_*o*_ and *i*_*o*_ at (*R*=50 Ω, *L*=50 mH), (b) *v*_*o*_ and *i*_*o*_ at (*R*=50 Ω, *L*=50 mH) to (50 Ω+100 mH), and (c) *v*_*C*1_, *v*_*L*_, *v*_*C*2,_ and *i*_*L*_ for (*R*=50 Ω, *L*=50 mH).

At a frequency of 20 kHz, switch *S*_1_ charges the inductor to 100 V, which is then discharged to the capacitors at a modulation index of 0.8. To provide the positive cycle, the switch *S*_3_ is turned on, and the voltage across the load will be 200 V. Fig 9(A) depicts the output voltage and current of the proposed inverter with a load value of 50+*j*50, a lagging power factor of 0.95, and a maximum load current of 3.0 A (*i*_*rms*_ =2.10 A). The reactive power should be controllable for most inverter applications with a lagging power factor. Changing the power factor from 0.95 to 0.85 demonstrates that the proposed inverter topology can function with a lagging power factor. The measured waveforms are depicted in Fig 9(B), which shows a minimum peak load current of 2.69 A. Fig 9(C) depicts the inductor along with the inductor current. These measured values demonstrate that the proposed inverter increases the input voltage by a factor of two when the modulation index is 0.8. In addition, this design controls all four switches with a single pulse generating technique, i.e., it does not require an additional pulse generator for boost operation, enhancing its efficiency.

**Fig 10:**
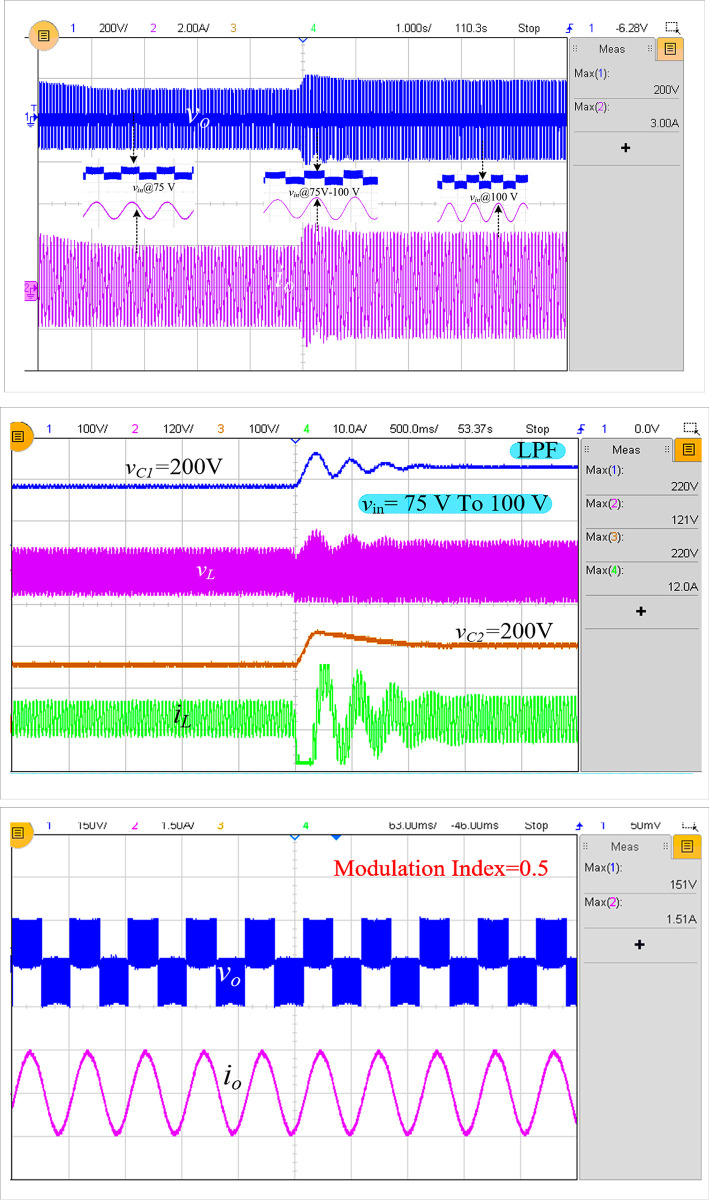
Experimental results in case of changing the input voltage from 75 V to 100 V: (a) *v*_*o*_ and *i*_*o*_, (b) *v*_C1_, *v*_*L*_, *v*_*C*2_, and *i*_*L*_ and (c) output voltage and current at a modulation index of 0.5.

Another crucial validation that must take place is a sudden change in the input, after which the switched/boost inverter must continue to operate and provide the same output voltage boosting ratio for a fixed duty cycle/modulation index. By increasing the input voltage of the suggested inverter from 75 V to 100 V, it was also tested. The measured voltage and current waveforms are shown in Fig 10(A), and the corresponding changes in the capacitor and inductor are shown in Fig 10(B).

**Fig 11:**
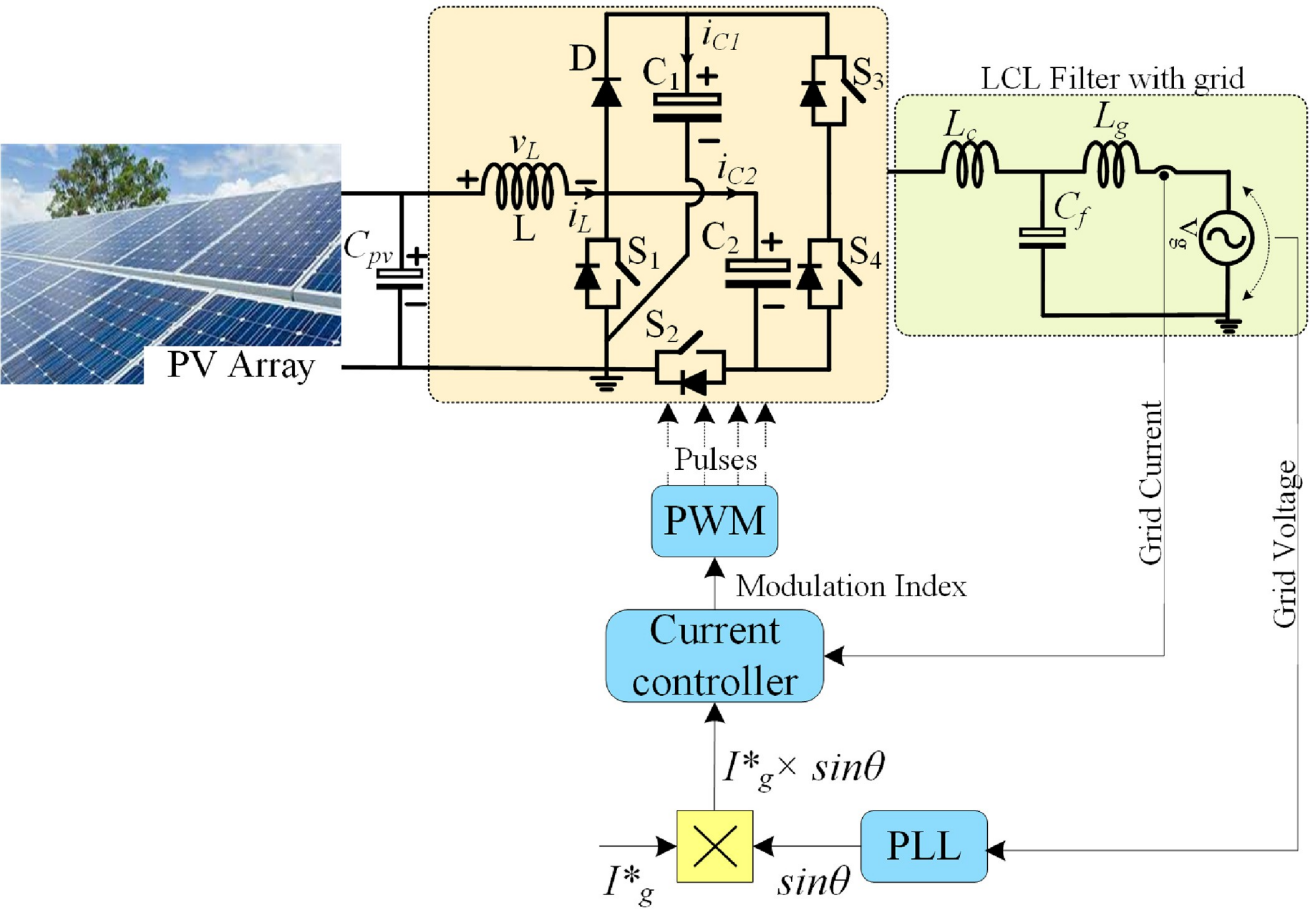
Control scheme for grid-tied PV system with the proposed inverter.

As shown in Fig 10(B), the inductor current and capacitor voltages oscillate to the maximum of 220 V capacitor voltage, and the maximum inductor current reaches 18 A. This occurs when there are sudden changes in the input. Fig 10(C) depicts the output voltage and current, which uses a reduced modulation index of 0.5. While decreasing the modulation index, the output voltage is dropped to 151 V, and the peak value of the maximum current, which is 1.51 A, is determined. Fig 11 depicts the overall control scheme used with a grid-tied system.

**Fig 12:**
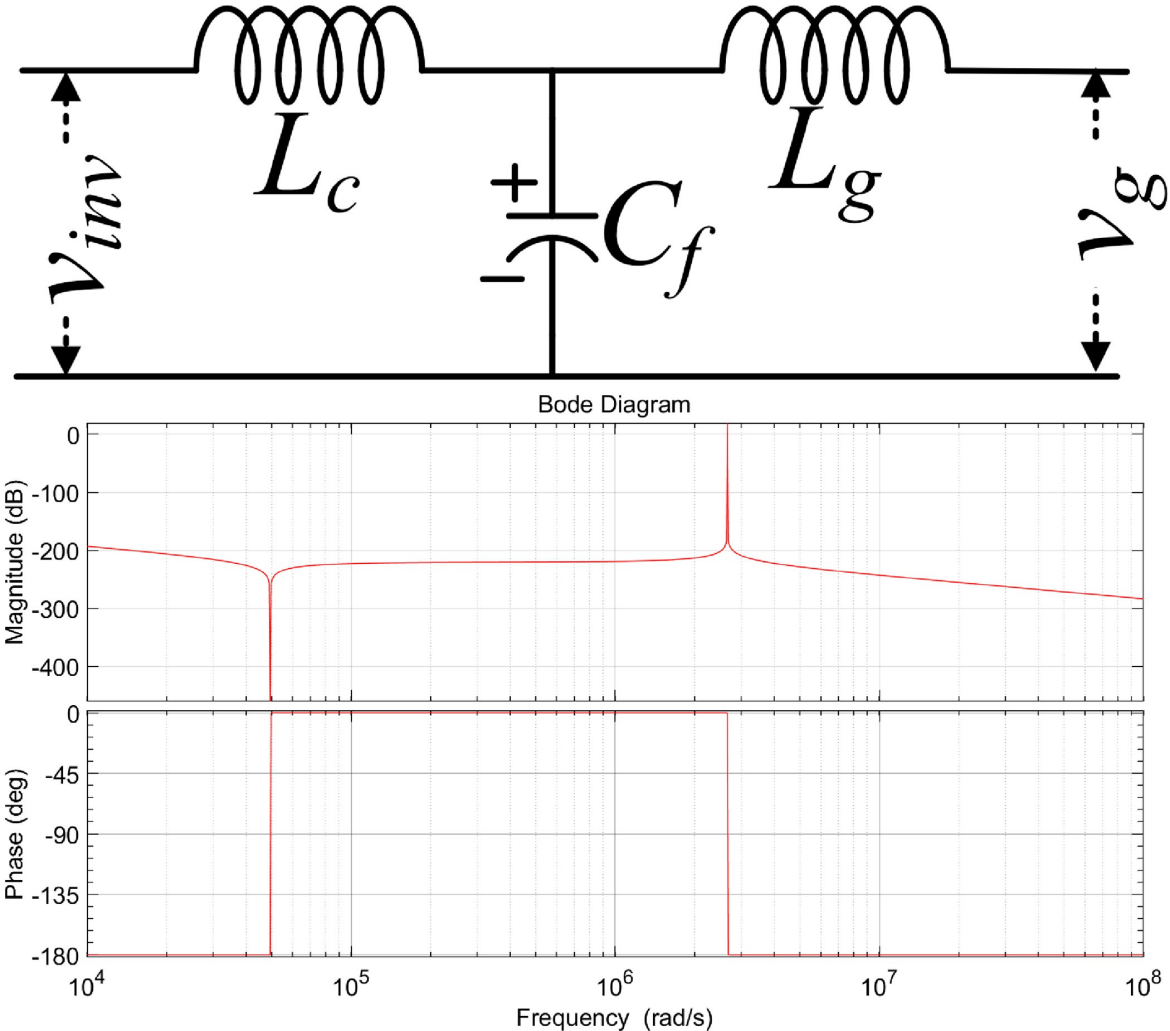
LCL filter design: (a) circuit diagram and (b) bode plot.

On the other hand, in the experimental setup, the direct DC source is used as the input rather than the PV, and MPPT tracking is not validated in this study. As can be seen in Fig 12, the LCL filter is placed in the path that runs between the proposed inverter output (*v*_*inv*_) and the grid (*v*_g_). A few assumptions are made to design the LCL filter, including setting the value of the lower LCL capacitor to three and assuming that the ripple attenuation (Δ*R*_*Iatt*_) is 20%. The LCL filter design values are given in Table 3 for 400 W output power with a fundamental frequency (*f*_*f*_) of 50Hz.

**Table 3:** LCL Filter design value.

*v*_*grms*_	*f*_*sw*_	*Z*_*b*_	*L*_*c*_	*C*_*f*_	*L*_*g*_	*f*_*res*_
		*V*_*g*_^2^ */P*_*o*_	*X*_*LC*_*/*2*πf*_*f*_	*C*_*max*_ */η*_*C*_	*r*×*L*_*C*_	4.5 kHz
110V/50Hz	20 kHz	30.25 Ω	7.5 mH	25 μF	16.4 μH	

where *Z*_*b*_ denotes the base impedance, *L*_c_ denotes the inverter side inductance, *C*_*max*_ denotes the maximum allowed capacitor value, *C*_*f*_ denotes the filter capacitance, *L*_*g*_ is the grid side inductance, and *r* is a function of the ripple attenuation. The LCL filter components value has been calculated as given below

$$L_{c}=\frac{v_{in}T_{sw}}{8\lambda_{c\_Lc}I_{c}}$$ (13)

$$C_{f}=\lambda_{c}\frac{P_{O}}{\omega_{o}v_{g}^{2}}$$ (14)

$$L_{g}=(\frac{1}{L_{c}C_{f}\omega_{h}^{2}-1})\times (L_{c}+\frac{\left|V_{inv}(j\omega_{h})\right|}{\omega_{h}\lambda_{h}I_{g}})$$ (15)

The transfer function of the LCL filter is given in (16), and its corresponding bode plot is shown in Figs 1 and 2(B).


$$LCLs=\frac{1}{L_{c}s}\frac{s^{2}+\frac{1}{L_{g}C_{f}}}{s^{2}+{\left(f_{res}2\pi \right)}^{2}}$$ (16)

**Fig 13:**
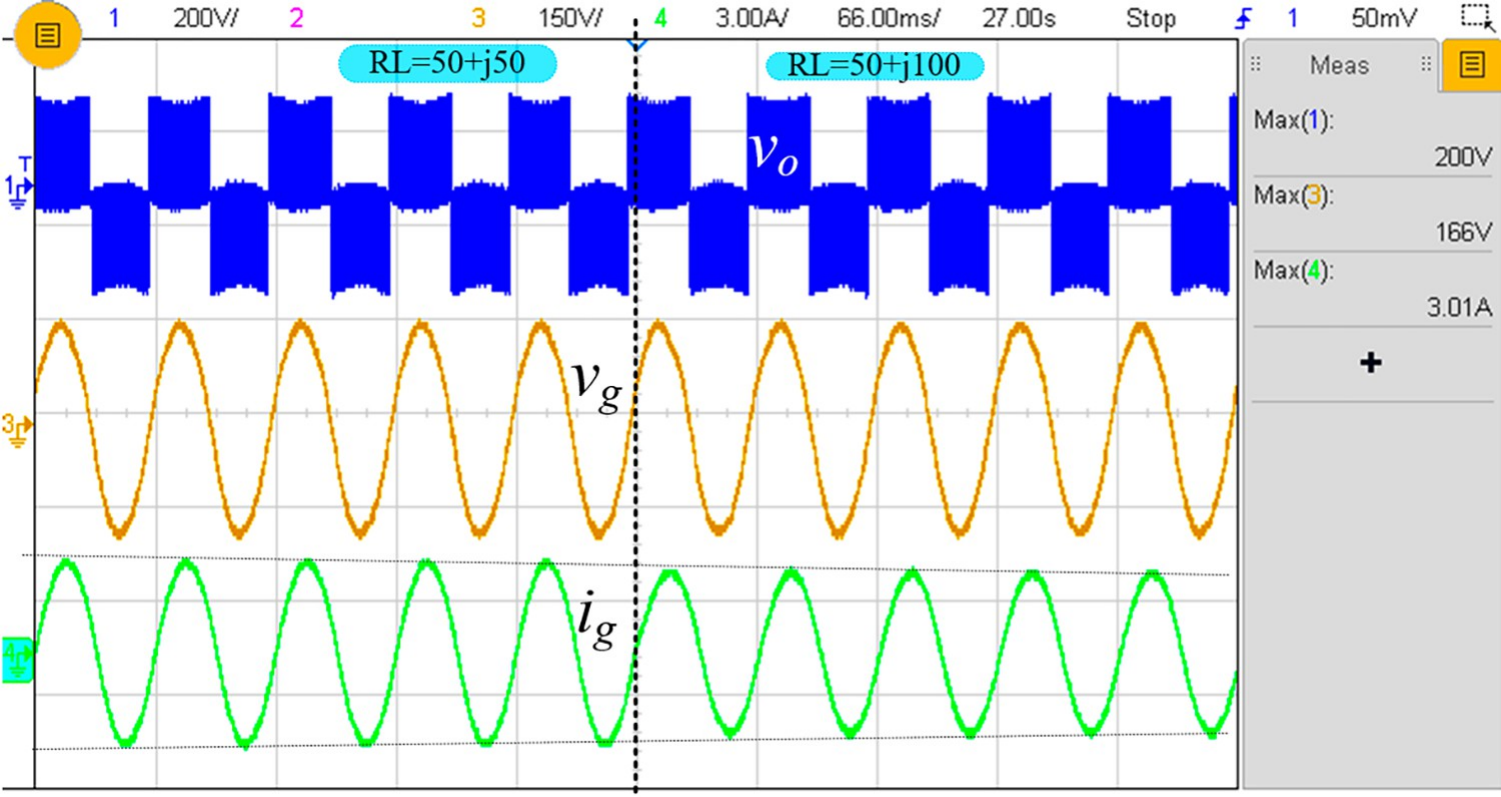
Experimental result of *v*_o_, *v*_g_, and *i*_g_ after LCL filter placement with load variation.

The proposed inverter is connected to the grid, and the corresponding inverter voltage, grid, and grid current are shown in Fig 13. The peak voltage of the grid is 160 V, equal to ~110V, and the grid current is also shown.

**Fig 14:**
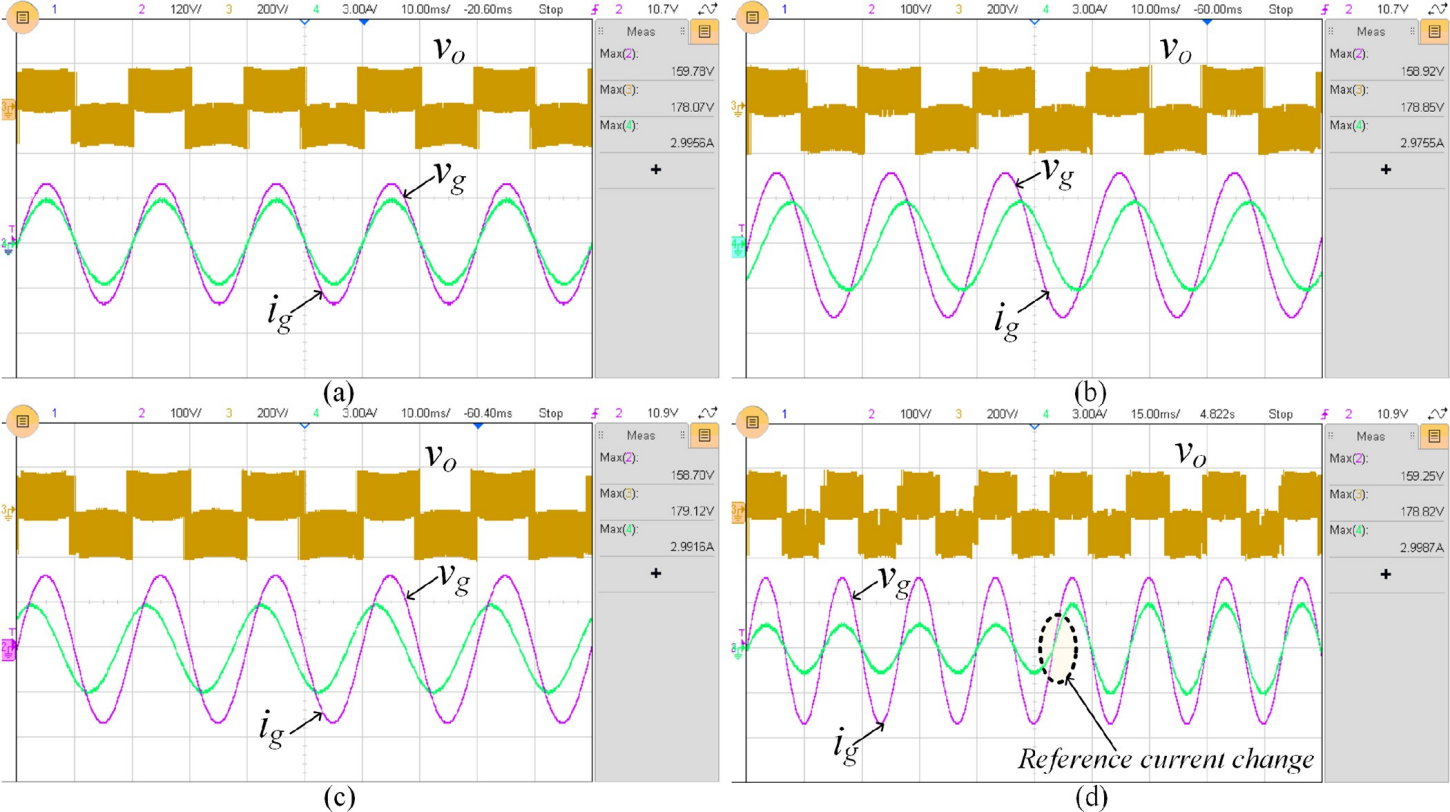
Experimental results of closed-loop (a) at unity PF, (b) at 0.71 lagging PF, (c) at 0.71 leading PF, and (d) during change of reference current (*i*_*g*, *ref*_) from 1.5 A to 3 A.

Furthermore, the proposed topology’s performance when connected to the grid under different power factors i.e., unity power factor, 0.71 lagging and leading power factor conditions is tested and the respective experimental results are shown in Fig 14(A)–14(C). The response while varying the grid reference (*i*_*g*, *ref*_) from 1.5 A to 3A is depicted in Fig 14(D).

In Table 4, a cost comparison is made between the proposed topology and existing single-stage, single-phase boost inverter topologies [10–14]. For the switches, ratings for 600 V are considered for all topologies, while the topology specifications determine the remaining passive components. The data in Table 4 confirm that the proposed topology costs less than the other alternative topologies.

**Table 4:** Cost comparison of the proposed topology with the existing topologies for *v*_*o*_=200 v.

Components	Model Number	Specification	Cost per piece ($)	This work		[11]		[10]		[14]		[12,13]	
				*TQ*	*TP*	*TQ*	*TP*	*TQ*	*TP*	*TQ*	*TP*	*TQ*	*TP*
IGBTs	IKFW50N60ETXKSA1	600V/50A	8.89	4	35.56	5	44.45	5	44.45	4	35.56	5	44.45
Gate driver	HCPL-316J-000E	IC = 150A, VCE = 1200V	6.69	2	13.38	3	20.07	3	20.07	2	13.38	3	20.07
Diode	FEP30GP-E3/45	400V/30A	2.95	1	2.95	-	-	1	2.95	-	-	1	2.95
Capacitor	ERLA211LIN172KA50M	250V/1700uF	8.27	2	16.54	1	8.27	2	33.08	1	8.27	1	8.27
	ALC70C102FP600	600V/1000uF	32.5	-	-	-	-	-	-	1	32.5	-	-
Inductor	Core Type	3mH/20A	12.46	1	12.46	1	12.46	-	-	-	-	1	12.46
		DTMSS-40/0.33/8V	4.24	-	-	-	-	1	4.24	0	0	0	0
		5mH/30A	18.24	-	-	-	-	-	-	1	18.24	-	-
Total price ($)				**10**	**80.89**	10	85.25	12	104.79	9	107.95	11	88.2

*TQ* denotes the total quantity, and *TP* denotes the total price.

**Fig 15:**
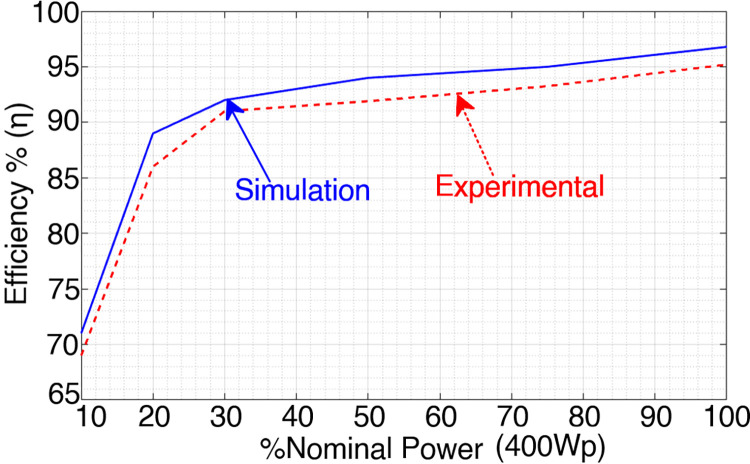
Efficiency variation for various power levels.

**Fig 16:**
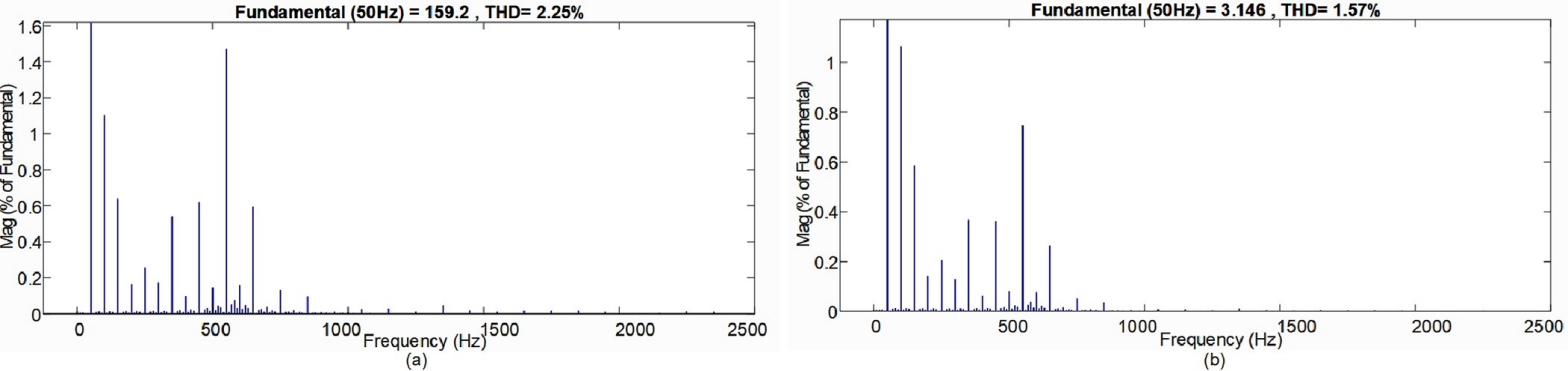
FFT analysis (a) Voltage THD (*V*_*THD*_), (b) Current THD (*I*_*THD*_).

Due to the reduced number of switches and gate drivers, the proposed inverter has a low cost. The efficiency of the proposed topology is computed with the PLECS simulation tools and measured with the experimental setup for resistive load, as shown in Fig 15. The power quality analysis of the proposed topology is carried out in the MATLAB simulation as can be seen from Fig 16, the voltage THD (*V*_*THD*_) and current THD (*I*_*THD*_) values of the proposed topology after the LCL filter while injecting the power to the grid is 2.25% and 1.57% which is within the limits of IEEE 519 standard for THD.

The simulated efficiency is 93.85%, while the actual efficiency is 92.2%. In addition, the maximum efficiency achieved in simulation is 98.15%, whereas the measured efficiency is ~97% for an output power of 400 watts.

## Conclusions

The paper presented a novel topology for single-phase, single-stage boost inverters, including a shared ground. In contrast to the topologies currently in use, the proposed topology employs a single diode and capacitor, reducing one switch along with its associated gate driver circuit. The extended structure demonstrates that the dual source is dual grounded, which presents a notable benefit of the suggested structure. This paper clarifies the design of the LCL filter utilized in the grid-tied system and verifies that the suggested topology is appropriate for reactive power regulation. Nevertheless, the efficiency of the inverter’s design was upheld at a reduced cost compared to alternative topologies. The suggested topology is most appropriate for the utilization of photovoltaic systems, particularly in the context of rooftop PV installations with limited scale. The experimental findings validate this claim, and the internationally accepted benchmark for efficiency was quantified and recorded.
